# Obesity-induced pancreatopathy in rats is reversible after bariatric surgery

**DOI:** 10.1038/s41598-018-34515-3

**Published:** 2018-11-02

**Authors:** Vinciane Rebours, Philippe Garteiser, Lara Ribeiro-Parenti, Jean-Baptiste Cavin, Sabrina Doblas, Gwenaël Pagé, André Bado, Alain Couvineau, Philippe Ruszniewski, Valérie Paradis, Maude Le Gall, Bernard E. Van Beers, Anne Couvelard

**Affiliations:** 1 Pancreatology Department, Beaujon Hospital, DHU Unity, AP-HP, Clichy, and Paris-Diderot University, Paris, France; 2 Inserm UMR1149, DHU Unity, and Paris-Diderot University, Paris, France; 3 General and Digestive Surgery, Bichat Hospital, AP-HP, and Paris-Diderot University, Paris, France; 4 Pathology Department, Beaujon Hospital, DHU Unity, AP-HP, and Paris-Diderot University, Paris, France; 5 Radiology Department, Beaujon Hospital, DHU Unity, AP-HP, Clichy, and Paris-Diderot University, Paris, France; 6 Pathology Department, Bichat Hospital, DHU Unity, AP-HP, and Paris-Diderot University, Paris, France

## Abstract

Obesity is a risk factor for pancreatic diseases. Bariatric surgery is one of the most efficient treatments of morbid obesity. The aims were to assess pancreatic endocrine and exocrine lesions in obese rats, to analyze effects of bariatric surgery. Sixty-three male Wistar rats were included in five groups: 2 fed with high fat diet (HFD) or normal diet for 3 months, 2 fed with HFD or normal diet for 6 months; 1 group fed with HFD and undergoing bariatric surgery (n = 30). Quantitative MR imaging was performed in HFD_6_, ND_6_ and HFD_3_-BS. Pancreas specimens were analyzed after sacrifice for adipocyte infiltration, fibrosis, acinar-ductal metaplasia, abnormality of Langerhans islets (HHF: hypertrophy, hypervascularisation, fibrosis), and hemosiderin deposits in acinar or endocrine locations. We found that HFD_6_ rats had more fibro-inflammatory islets (P = 0.0139) and acinar-ducal metaplasia (P = 0.0843) than HFD_3_ rats. Rats with HFD_3+6_ had more fibro-inflammatory islets (P < 0.0001), hemosiderin deposits (p < 0.0001), fat infiltration (P = 0.0008) and acinar-ductal metaplasia lesions (P = 0.0424). Weight increase was associated with glycoregulation abnormalities (r = 0.44, P = 0.08) and adipocyte infiltrations (P = 0.009). After surgery, less fibro-inflammatory islets (P = 0.0004), fat and iron infiltrates (P = 0.005 and P = 0.06), and acino-ductal metaplasia (P = 0.05) were observed compared to HFD_6_ rats. MR image quantifications revealed increased elasticity, fat fraction, and R2 and a decreased elasticity wave dispersion coefficient in the high fat groups that reversed after surgery. MRI parameters were in strong correlation with respective histological counterparts. In conclusion, obese rats develop pancreatic inflammatory lesions with acinar-ductal metaplasia in acinar location and the endocrine-exocrine interface. These changes can be prevented by bariatric surgery. Quantitative MR imaging is accurate in identifying early pancreatic lesions.

## Introduction

Chronic pancreatitis is a complex disease, usually due to the exposure to several risk factors. Chronic alcohol intake was previously considered as the main environmental cause in Western countries. Since a decade ago, tobacco consumption and genetic determinism have been well established as independent risk factors^1^. The role of obesity in the spectrum of pancreatic diseases is still controversial. In 2014, a meta-analysis of factors predisposing to pancreatic diseases (acute and chronic pancreatitis and pancreatic cancer) in the general population included 51 population-based studies with more than 3 million individuals and nearly 11,000 patients with pancreatic diseases. Tobacco use was the most important risk factor for pancreatic diseases (relative risk (RR), 1.87; 95% confidence interval (CI), 1.54–2.27), followed by obesity (RR, 1.48; 95% CI, 1.15–1.92) and heavy use of alcohol (RR, 1.37; 95% CI, 1.19–1.58)^2^. Numerous epidemiological studies confirmed that obesity is a risk factor of pancreatic cancer in obese men and women (Body mass index (BMI), kg/m^2^ ≥30.0), with a relative risk estimated to 1.76 (95% CI, 0.90–3.45) and 1.70 (95% CI, 1.09–2.64), respectively^3^. Multiple mechanisms are involved, including pro-inflammatory cytokines secreted by the adipose tissue, insulin resistance, and hyperinsulinism. In acute pancreatitis, obesity is a primary risk factor for developing local complications (abscess, pseudocyst, necrosis), organ failure, and death. Meta-analysis of the published studies reported a relative risk of 4.3 for local complications, 2.0 for systemic complications, and 2.1 for death^4^.

The link between obesity and pancreatic inflammation remains controversial. No epidemiological series have confirmed that obesity is an independent risk factor of chronic pancreatitis. However, in 2010, Ammann *et al*. hypothesized that obesity before alcohol chronic pancreatitis onset might be an additional susceptibility factor^5^. In 227 patients with chronic alcoholic pancreatitis, 54.2% were overweight and 15% were obese before disease onset versus 37.7% and 3.1% in a control group, respectively. In this series, the highest BMI before disease onset did not impact on some major variables such as outcome of exocrine insufficiency, diabetes, calcification, and mortality from chronic pancreatitis; however, being overweight before disease onset appeared to be a risk factor for chronic pancreatitis.

Increasing evidence suggests that fat-derived mediators may interfere with inflammatory and immune responses. Obesity is associated with chronic low-grade inflammation (also called meta-inflammation), characterized by abnormal pro-inflammatory cytokine production, immune activation, disruption in autophagy, promotion of endoplasmic reticulum stress, mitochondrial dysfunction, and increased inflammatory signaling. Obesity increases the size of adipocytes, leading to their necrosis and a significant accumulation of activated macrophages^6,7^. The inflammatory mediators, including IL-6, IL-1, and TNF-α, have been implicated in the development of insulin resistance and their levels increase within adipose tissue. This inflammation is accompanied by up-regulation of anti- inflammatory factors, such as IL-10, IL-4, and TGF-β. The inflammatory mediators secreted by macrophages act not only locally, in a paracrine manner, but also contribute to systemic inflammation^8,9^. All together, these data suggest that obesity might be an independent risk factor of chronic pancreatopathy.

Preliminary studies have shown the feasibility and potential diagnostic value of quantitative magnetic resonance (MR) imaging for evaluating the normal and diseased pancreas. These methods include MR elastography for assessing the degree of pancreatic fibro-inflammation, chemical shift-encoded multi-echo gradient-echo imaging for assessing the proton density fat fraction (PDFF) and transverse relaxation rate (R2*, proportional to iron concentration) and diffusion weighted-MR imaging with apparent diffusion coefficient (ADC) measurements for assessing pancreatic fibrosis and cellularity^10–14^.

The aims of this study were to characterize pancreatic endocrine and exocrine lesions in obese rats compared to a control group with pathological examinations and quantitative MR imaging and to assess the evolution of these lesions after bariatric surgery.

## Methods

### Animal groups

All animal use conformed to the European Community guidelines and was approved by the local ethics committee (Comité d’Ethique Paris-Nord (no. N°121), 02285.03) and the Ministry of Higher Education and Research (N° APAFIS#8290-2016122015078962).

Five groups of 5-week-old, male Wistar rats, out-bred strains, weighing 220–240 g were assigned as follows:

- Two groups of rats were fed with high-fat diet (HFD) (Altromin C1090) either for 3 months (group HFD_3_, n = 9) or for 6 months (group HFD_6_, n = 9), with their respective control groups fed with a normal diet (ND) (Altromin 1324) for 3 months (group ND_3_, n = 9) or 6 months (group ND_6_, n = 6).
- Additionally, two surgical groups of rats fed with HFD for 3 months (group HFD_3_-BS, n = 30) were constituted, one operated by Roux-en-Y gastric bypass (GP) (group: HFD_3_-BS/GP; n = 19) or one the other by sleeve gastrectomy (SG) (group: HFD_3_-BS/SG; n = 11). Surgery were performed as previously described by our team and rats were sacrificed up to 3 months after the surgery^15^.

Weight and blood glucose level before sacrifice were assessed. Glycemia was measured with the AccuChek System (Roche Diagnostics, Meylan, France) and expressed in mg/dL.

### Animal bariatric surgery and post-surgery procedures (group HFD3-BS)

Male Wistar rats fed with high-fat diet for 3 months underwent BS, either GB (group HFD_3_-BS/GB) or SG (group HFD_3_-BS/SG). They were fasted overnight before operation. Anesthesia was induced by intraperitoneal injection of pentobarbital (Ceva, Libourne, France). Standard aseptic procedures were used throughout. After laparotomy, the stomach was isolated outside the abdominal cavity. Loose gastric connections to the spleen and liver were released along the greater curvature and the suspensory ligament supporting the upper fundus was severed.

#### Roux-en-Y Gastric Bypass (GB)

The forestomach was resected using an Echelon 45-mm staple gun with blue cartridge (Ethicon, Issy les Moulineaux, France). Then the gastric pouch was created with a TA-DST 30-mm-3.5-mm stapler (Covidien, Courbevoie, France), preserving the arterial and venous supply. The jejunum was transected 15 cm distally from the pylorus. The Roux limb was anastomosed to the gastric pouch and the biliopancreatic limb was anastomosed 20 cm distally to gastrojejunal anastomosis with 6-0 polydioxanone running sutures.

#### Vertical Sleeve Gastrectomy (SG)

After resection of the forestomach as described, 80% of the stomach was resected with an application of Echelon 45-mm staple gun, leaving a thin gastric tube in continuity with the esophagus and keeping the antrum in place.

### Histological and immunohistochemical examination of pancreas

#### Tissue preparation and staining

Entire pancreatic specimens were fixed in formalin and included in paraffin after sacrifice for all groups of rats. All hematoxylin-eosin–safran (HES)-stained slides of pancreas were analyzed by two investigators (AC and VR) for each case, both blinded to the group status. The number of blocks available per specimen depended on the size of the pancreas (1 or 2 blocks). We selected one block in each case for the final pathological analyses and calculated the total surface area of the pancreas for that block in cm^2^. Specific stains (Picrosirius-Hemalun, Perls) and immunohistochemical analyses were performed on one slide each, serially cut from this selected block.

#### Evaluation and scoring

The HES, Picrosirius-Hemalun, and Perls stainings were evaluated by two investigators (AC and VR) blinded to both clinicopathological and outcome data of animals. Fibrosis, inflammation, acino-ductal metaplasia, fat infiltration, hemosiderin deposits and modifications of islets were independently assessed.

The number of fibro-inflammatory foci per specimen were evaluated on Picrosirius-Hemalun staining. The fatty infiltration was assessed on HES staining by the count of intrapancreatic adipocytes per specimen. Acino-ductal metaplasia, assessed on HES, was defined as the replacement of acinar cells by duct cells, a switch from differentiated acinar cells to differentiated ductal cells. Hemosiderin deposits were counted on Perls stain. Islets were considered as abnormal when they were hypertrophic, hyper-vascularized, fibrotic, and disorganized or poorly delineated. These modifications were called HHF lesions. The number of HHF lesions was recorded on HES staining. For all these modifications (number of fibro-inflammatory and acinar-ductal metaplasia foci, number of adipocytes, number of hemosiderin deposits, and number of HHF lesions), a ratio of the number of lesions divided by the total pancreatic surface area analyzed was calculated.

#### Immunohistochemistry

Five µm sections were obtained from each selected block of pancreas. Immunohistochemistry was performed with an automated immunohistochemical stainer according to the manufacturer’s guidelines (Streptavidine-peroxidase with an automate Bond III Leica, Germany). Immunostaining was performed after the slides were dewaxed and rehydrated. Antigen retrieval was conducted by pretreatment at a high temperature at pH9 in TRIS Buffer. PBS was substituted for the primary antibody and used as a negative control. The slides were immunolabeled with monoclonal or polyclonal antibodies against insulin/β cells (Biorad, USA, 1:25, ref 171B7008M), CD34/endothelial cells (Abcam, 1:2000, ab81289), alpha-smooth muscle actin/activated stellate cells (Abcam, 1:200, ref ab5694), CD68/macrophages (Abcam, 1:400, ab125212), Cytokeratin 7/pancreatic ducts (Abcam, 1:8000, ab181598) and CD 45/lymphocytes (Abcam, 1:300, ab10558) in order to analyse the islets, the microvessels, the infiltration by macrophages and lymphocytes, and the activated stellate cells.

#### Morphometry by software image analysis

Slides stained with HES, Masson’s Trichrome and Perls were scanned with a computer-controlled capture device (Aperio; Leica Biosystems, Nanterre, France). We used the PRECISION image analysis software (Aperio) for the quantification of total pancreatic surfaces (HES), as well as for the quantification of fibrosis (Masson’s Trichrome), hemosiderin deposits (Perls), and islets surface (immunohistochemistry with insulin antibody) as a % of the total surface area.

### Imaging procedure

MR elastography and MR imaging of the entire pancreas were performed just after sacrifice for the following rats: HFD_6_, n = 6; ND_6_, n = 8 and HFD_3_-BS (high fat diet for 3 months followed by by-pass surgery), n = 5. All rats were randomly selected from their group.

The pancreas was dissected from the surrounding intraperitoneal adipose tissue and maintained in physiological serum. Pancreas explants were immobilized on a custom-made MR elastography actuating cradle^16^, submerged in medium and maintained at 37 °C for the duration of the experiments. The proper positioning of the mechanical transducer was verified using a conventional T1-weighted gradient echo sequence acquired with and without a fat saturation pulse.

#### MR elastography

MR elastography was performed using a spin-echo echoplanar sequence with motion encoding gradients used sequentially along the three directions. To accommodate the softness of pancreatic tissue^17^, relatively low frequencies of 400 Hz, 600 Hz, and 800 Hz were selected, and each frequency was assessed at 4 mechanical offsets per cycle. Acquisition parameters were TR/TE of 1000 ms/18 ms, allowing 1, 2, or 6 periods of motion encoding respectively for the three probed frequencies, 300 µm isotropic resolution, one average and a readout bandwidth of 25 kHz. Parametric maps of elasticity (storage modulus G’) and its wave dispersion coefficient or frequency dependence (γ) expressed as the exponent of a fit to a power law were obtained by algebraic inversion of the wave propagation equation after having applied the curl operator to the unwrapped shear component of the wave displacement fields^14,16,18^. Due to a technical failure, one pancreas in the HFD_6_ group could not be examined with MR elastography.

#### Fat fraction and R2*

A chemical shift-encoded gradient-echo MR imaging sequence with 15 consecutive echoes (first TE of 1.65 ms, subsequent echo train spacing of 0.92 ms) was used to calculate the pancreatic fat fraction and R2*. The other parameters were in-plane resolution of 250 µm, 1 mm slice thickness, 300 kHz bandwidth, 950 ms TR, 15° flip angle, and 4 signal averages. Parametric maps of fat fraction and R2* were extracted by fitting the magnitude images to a model accounting for transverse relaxation time (T2*) decay, noise, and signal interferences between water and the seven most abundant lipid peaks^19–21^.

#### Diffusion-weighted MR imaging

Diffusion-weighted MR imaging was performed with a spin-echo sequence and 21 segment echoplanar readout, 4 b-values (0, 150, 300, 500, and 700 s/mm²), 195 µm in-plane resolution, 400 µm slice thickness, 2000 ms/20 ms TR/TE, a readout bandwidth of 250 kHz and 3 signal averages. The apparent diffusion coefficient (ADC) was calculated on a pixel-by-pixel basis using custom made software running the root framework (ROOT, v5.34.01; CERN, Geneva, Switzerland). As the images concerned *ex vivo* explants, no perfusion component was expected, hence the fitted equation was S(b_i_) = S_0_ × exp(−ADC × b_i_), with S(b_i_) the signal obtained at the i-th b value, S_0_ the signal in absence of diffusion weighting, ADC the apparent diffusion coefficient and b_i_ the various b values. ADC and S_0_ were kept as free parameters.

#### Regions of interest

Regions of interest were selected on the MR elastography maps. The regions of interest were positioned at the center of the pancreas, while avoiding the MR elastography piston transducer. The extent of the regions of interest was maximized under the constraint of having mechanical wave amplitudes larger than 8 µm. This step was taken to ensure accuracy of the MR elastography results by providing sufficiently strong wave amplitudes for proper inversion of the wave equation. Regions of interest defined with this procedure were then reported onto the other parametric maps with appropriate geometric transformations to accommodate the various fields of view and resolutions.

### Statistical analysis

General characteristics were expressed as medians and ranges, or percentages. Comparisons of general and morphological characteristics, MR, and pathological data between groups were performed using the Mann-Whitney test or the Kruskall–Wallis test and Dunn post-test for continuous data and with the Chi2 test or the Fisher’s exact test for categorical data. Spearman correlation coefficients were used to search for correlations between continuous variables.

Data were analyzed with the SAS 9.1 statistical software for Windows (SAS Institute Inc., Cary, NC) and MedCalc Statistical Software version 17.6 (MedCalc Software bvba, Ostend, Belgium; http://www.medcalc.org; 2017). All statistical tests were two-sided. The critical level of statistical significance was set at p < 0.05.

## Results

**Figure 1:**
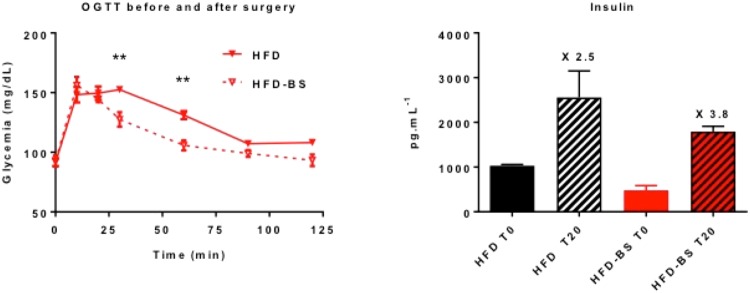
Oral glucose tolerance test (OGTT) and insulin secretion levels before and after bariatric surgery. After bariatric surgery, the glucose tolerance OGTT and insulin secretion improve in rats.

Main characteristics and pathological lesions of the five groups are summarized in Table 1. Acinar-ductal metaplasia lesions were correlated to iron deposits (r = 0.649 (0.5429–0.7347), P < 0.0001), HHF lesions (r = 0.7412 (0.6562-0.807), P < 0.0001) and fat infiltration (r = 0.193 (0.027–0.035), P = 0.0197). Iron deposits were associated with HHF lesions, r = 0.8276 (0.7672–0.8734), P < 0.0001 and fat infiltration, r = 0.5955 (0.4733–0.6952), P < 0.0001. After bariatric surgery, the oral glucose tolerance and insulin secretion levels improve in rats. Figure 1.

**Table 1:** Characteristics of the rats: type of diets, morphological and pathological data.

Type of Diet	HFD_3_ N = 9	HFD_6_ N = 9	ND_3_ N = 9	ND_6_ N = 6	HFD_3_-bariatric surgery N = 30
	HFD	HFD	ND	ND	HFD
Duration of diet before sacrifice (months)	3	6	3	6	3
Weight before sacrifice (gram)*	595 [464–695]	817 [697–1083]	521 [494–626]	585 [538–644]	579.5 [488–692]
Number of fibrosis and ADM spots*^§^	0.41 [0–0,84]	0.95 [0–5]	0 [0–1.85]	0 [0–1.3]	0 [0–4.7]
Number of HHF lesions*^§^	2.05 [0–6.28]	6.05 [2.88–21.7]	0 [0–0]	0 [0–2]	0.9 [0–10.8]
Number of iron deposits*^§^	3.4 [0–6.28]	4.43 [0–21.75]	0 [0–0.4]	0 [0–1.3]	1,3 [0–19]
Number of adipocytes*^§^	34.7 [5.7–119.3]	65.35 [1–141.8]	15.5 [0–27.7]	11 [0–34.6]	10.7 [0–66.7]

HFD, high fat diet; ND, normal diet; ADM, acinar-ductal metaplasia; HHF lesions: hypertrophy, hyper-vascularization and fibrosis involving islets.

*Expressed as median and range per specimen.

^**§**^Data expressed as a ratio: number of lesions per cm^2^.

### Description of HFD rats

#### Description of pancreas pathology in HFD rats

**Figure 2:**
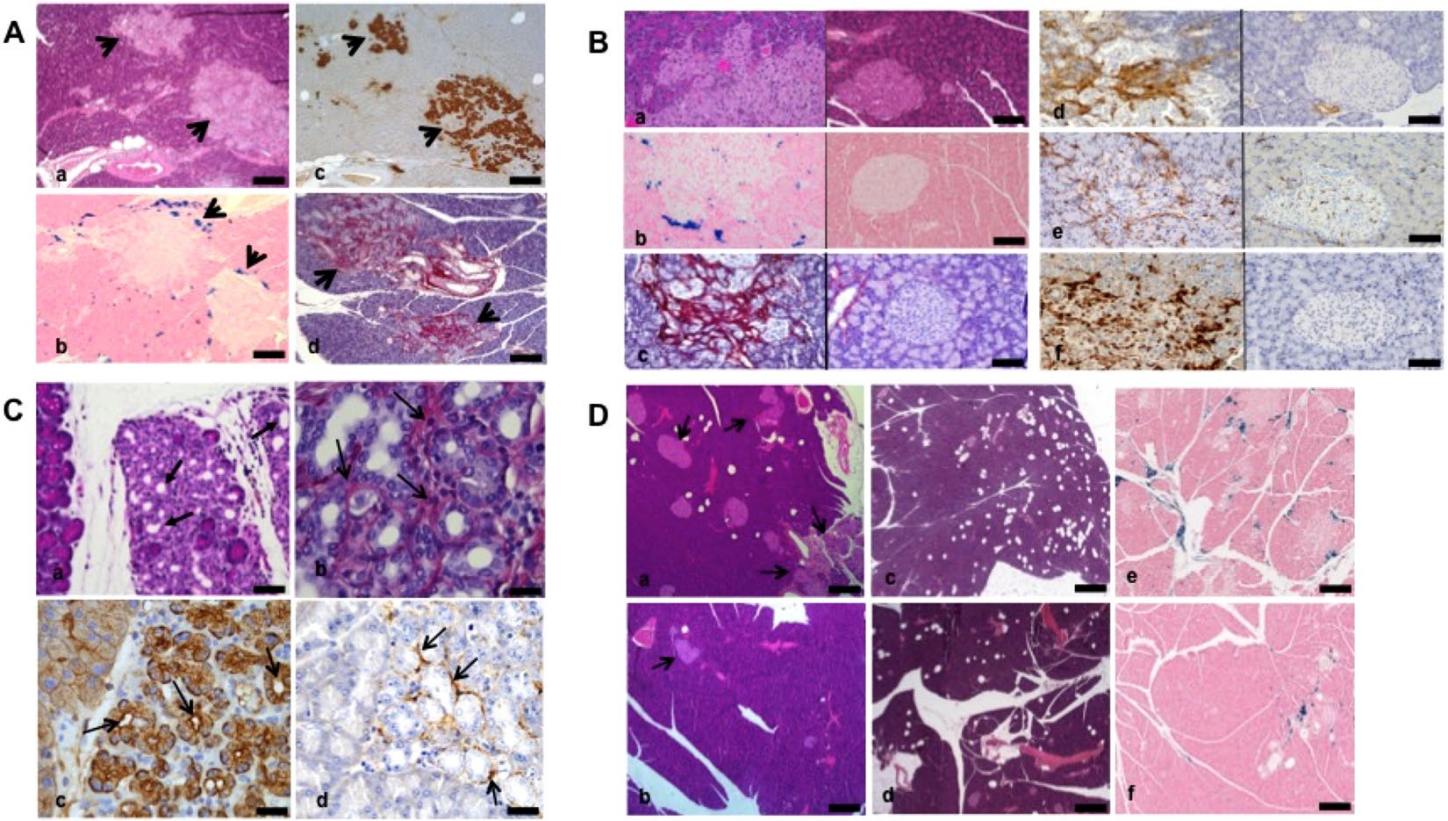
(**A**) Modifications of the pancreatic parenchyma in obese rats. Numerous HHF lesions are scattered and heterogeneously distributed among pancreatic parenchyma (a–c). They are associated with hemosiderin deposits, colored in blue, mainly distributed at the interface between exocrine and endocrine areas but also at a distance from HHF (b). HHF are heterogeneous in shape and size containing neuroendocrine insulin-secreting cells, highlighted on insulin staining (c). They are fibrous on picrosirius stain, the fibrous scare largely invading the surrounding exocrine tissue (d). HHF: modified islets hypertrophic, hypervascularized and fibrotic. (a) HPS staining; (b) Perls staining; (c) immunostaining with insulin antibody; d) Picrosirius-hemalun staining. Scale bars: 200 µm (a–d). (**B**) Morphology of HHF in pancreas of obese rats (left images) compared to normal diet rats (right images) In obese rats, HHF are large poorly delineated structures extending from remodeled islets into the surrounding exocrine component, as compared to small well-delimited normal islets (a). They are accompanied by abundant hemosiderin deposits colored in blue by Perls staining mainly found at the interface with the exocrine tissue, and also at a distance at a lesser extent; normal pancreas do not contain any hemosiderin deposit (b). HHF contain large amounts of fibrosis highlighted by picrosirius stain (c) largely invading the exocrine pancreas between adjacent acinar cells; in contrast non-obese islet do not contain any detectable amount of fibrosis. HHF include many activated stellate cells expressing smooth muscle actin (d) and present an increased vascularization, as highlighted by CD34 immunostaining (e), as compared to normal islets. HHF are infiltrated by numerous inflammatory cells, including lymphocytes (f) and macrophages. In normal non-obese rats, small islets and surrounding exocrine parenchyma do not contain any infiltrating macrophages or lymphocytes HHF (modified islets, hypertrophic, hypervascularized and fibrotic) in obese rats (left images) and pancreas of normal diet rats (right images). (a) HPS staining; (b) Perls staining; (c) Picrosirius-hemalun staining. (d) immunostaining with a smooth muscle actin antibody; d) immunostaining with CD34 antibody; (d) immunostaining with CD45 antibody. Scale bars: 100 µm (a–f). (**C**) Morphology of exocrine modifications in obese rats (acino-ductal metaplasia and fibrosis) Areas of acino-ductal metaplasia (arrows) are located in the exocrine parenchyma(a). In these areas, the acinus are dedifferentiated and form small tubular structures as ducts (a,b). These exocrine areas are fibrotic, transformed acinar cells are separated by bands of fibrosis highlighted by picrosirius stain (b, arrows) and strongly stained with CK7 antibody (c, arrows) as compared to the faint staining on normal acinus on the left. Activated pancreatic stellate cells, stained with anti-smooth muscle actin antibody, are located in areas of acino-ductal metaplasia (d). (a) HPS staining; (b) Picrosirius-hemalun staining; (c) Immunostaining with CK7 antibody; (d) immunostaining with a smooth muscle actin antibody. Scale bars: 100 µm (a); 30 µm (b); 50 µm (c,d). (**D**) Comparison of pancreatic morphology between obese rats before (a,c,e) and after bariatric surgery (b,d,f) Modified hypertrophic, hypervascularized and fibrotic islets (arrows), areas of acinar fibrosis, adipocyte infiltration and iron deposits are less prominent in the pancreas of operated rats (b,d,f) as compared to obese non-operated rats (a,c,e). The modified islets, infiltration of adipocytes and deposits of iron are heterogeneous from one to other area in the same pancreas. (a–d) HPS staining; (e,f) Perls staining. Scale bars: 500 µm (a,b,e,f); 1000 µm (c,d).

HFD rats presented numerous scattered HHF lesions: some islets were hypertrophic, hyper-vascularized, fibrotic and disorganized, poorly delineated, and surrounded by extensive fibrosis and inflammatory infiltrate spreading into the surrounding exocrine parenchyma. Inflammatory cells included numerous macrophages and lymphocytes. HHF lesions were associated with iron deposit around and into islets, especially in case of important fibrosis infiltration; irons deposits were also noted in exocrine parenchyma at a distance from islets. Adipocyte infiltration was observed in intra and extra lobular locations. In the exocrine parenchyma, acinar-ductal metaplasia was observed in areas of fibro-inflammatory foci; containing macrophages and lymphocytes and activated pancreatic stellate cells (Fig. 2A–C).

#### Comparison between HFD_3_ and HFD_6_

**Figure 3:**
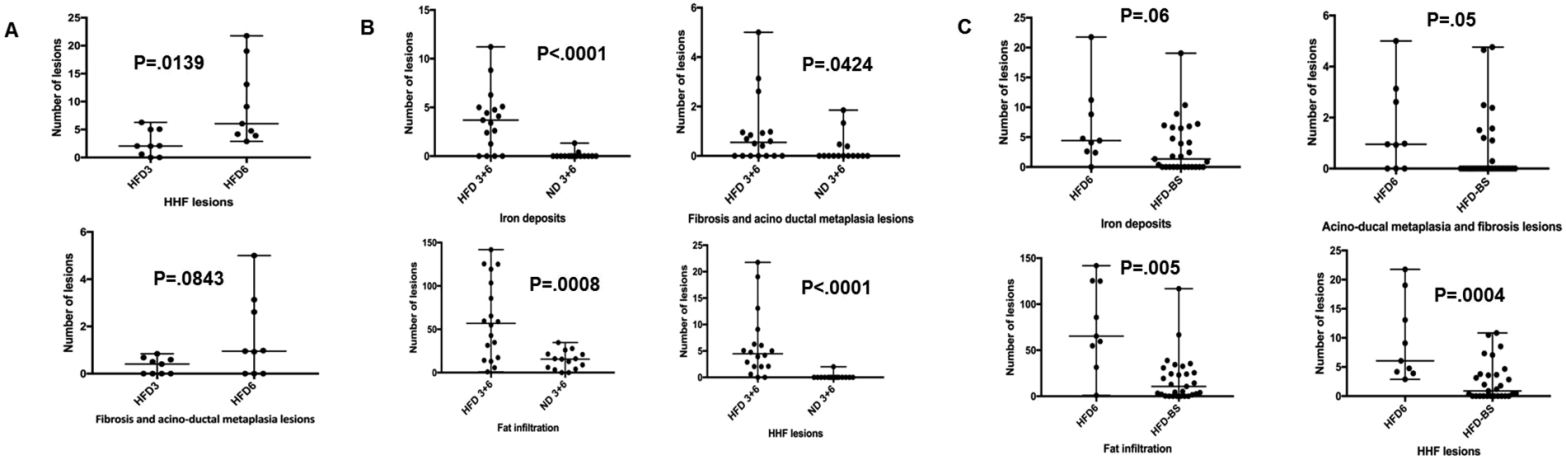
HHF lesions of the islets, acinar-ductal metaplasia lesions, hemosiderin deposits and adipocyte infiltration (**A**) in the HFD_3_ and HFD_6_ groups, (**B**) in the HFD groups (HFD_3_ and HFD_6_) and in the control groups (ND_3_ and ND_6_), (**C**) in the HFD_6_ and the bariatric surgery groups.

The median weight before sacrifice was higher in the group *HFD*_6_ compared to *HFD*_3_ (817 g [697–1083] versus 595 [464–695]), P = 0.0005. Older rats presented more HHF lesions, P = 0.0139. Foci of fibrosis with acino-ductal metaplasia lesions were quite significantly more found in HFD_6_, P = 0.0843. The median glucose blood level was higher in the group *HFD*_6_, P = 0.0063. Adipocyte infiltration (P = 0.136) and iron deposits (P = 0.23) did not significantly differ between the two groups. Figure 3A.

#### Comparison of controls: ND_3_ versus ND_6_ rats

No significant difference was found between the 2 groups regarding the number of fibrosis foci, iron deposits, acino-ductal metaplasia lesions, adipocytes (fat infiltration), and HHF complexes. The weight before sacrifice was not significantly different.

#### Comparison between HFD rats (HFD_3_ and HFD_6_) and controls (ND_3_ and ND_6_)

The comparison between HFD and ND rats showed an increase of HHF lesions (P < 0.0001), hemosiderin deposits (P < 0.0001), adipocyte infiltration (P = 0.0008), and acino-ductal metaplasia lesions and fibrosis (P = 0.0424) in HFD rats. The increase of the weight in HFD rats was associated adipocyte infiltration (P = 0.009). In the 4 groups, HHF lesions were associated with hemosiderin deposits (r = 0.88, P < 0.0001). Figure 3B.

### Role of Bariatric surgery on HFD rat lesions

In the surgery group, no difference was found regarding morphological lesions according to the type of procedures (by pass or sleeve gastrectomy).

#### Comparison of pathological data between HFD rats and HFD-Bariatric surgery

An analysis was performed in HFD rats after bariatric surgery compared to HFD rats fed during 3 months. In rats operated on, the number of adipocytes (P = 0.017) drastically decreased. However, HHF, acino-ductal metaplasia and iron deposits were not different between the 2 groups. When the analyses were conducted between the HFD_6_ and HFD-bariatric surgery groups, operated rats presented less HHF lesions (P = 0.0004), fat infiltrate (P = 0.005), and acino-ductal metaplasia lesions (P = 0.05). Figures 2D and 3C.

### MR imaging in control, obese and surgery groups

Imaging procedures were performed in rats from the obese group (HFD_6_) weighing 863 ± 141 g, the control group (ND_6_) weighing 586 ± 42 g, and the bariatric surgery group weighing 640 g [508–680].

#### MR elastography

**Figure 4:**
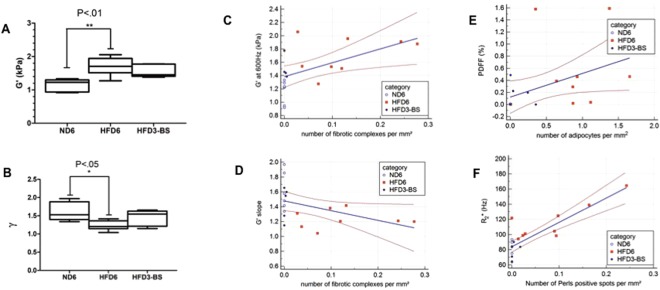
(**A**) Boxplots of elasticity (G’) in control rats, obese rats and rats after surgery. Obese animals have higher elasticity than controls. The elasticity is lower after surgery than in obese rats. Boxes extend from the 25^th^ to the 75^th^ percentiles, central lines indicate median values, and whiskers extend from the 2.5^th^ to the 97.5^th^ percentiles. (**B**) Boxplots of wave dispersion coefficient (γ) at MR elastography in control rats, obese rats and rats after surgery. Frequency dispersion is significantly decreased in obese animals, and has an intermediate value in the surgical group that is indistinguishable from either the control or the obese group. Boxes extend from the 25^th^ to the 75^th^ percentiles, central lines indicate median values, and whiskers extend from the 2.5^th^ to the 97.5^th^ percentiles. (**C**) Scatter diagram of elasticity against number of HHF. The regression line for all samples (ND rats: hollow circles, obese rats: plain squares, rats after surgery: plain circles), is depicted (solid line) with 95% confidence intervals (dotted line). Significant positive correlation is found between the elasticity and histopathologically determined fibrosis marker (p = x, r = y). (**D**) Scatter diagram of wave dispersion coefficient (γ) against number of HHF. The regression line for individuals of all groups (ND rats: hollow circles, obese rats: plain squares, rats after surgery: plain circles), is depicted (solid line) with 95% confidence interval (dotted line). Significant negative correlation is found between wave dispersion coefficient and histopathologically determined fibrosis marker (p = x, r = y). (**E**) Scatter diagram of MR imaging derived pancreatic proton density fat fraction (PDFF) against number of adipocytes. The regression line for the individuals of all groups (ND rats: hollow circles, obese rats: plain squares, rats after surgery: plain circles) is depicted (solid line) with 95% confidence intervals (dotted line). Significant correlation is observed between the MR imaging marker and the pathological fat infiltration (p = x, r = y). (**F**) Correlation of R_2_* measurements with the number of Perls positive spots expressed per unit area. The regression line for individuals of all groups (ND rats: hollow circles, obese rats: plain squares, rats after surgery: plain circles) is depicted (solid line) with 95% confidence intervals (dotted line). Significant correlation is observed between R2* and Perls positive spots.

The elasticity was significantly different between normal, obese, and surgery groups (1.23 [0.92–1.33] kPa in normal rats versus 1.71 [1.27–2.06] kPa in obese rats and 1.46 [1.38–1.78] kPa after surgery, P = 0.0037). Post-hoc tests showed that the storage modulus was significantly higher in obese rats versus controls (P < 0.01). After surgery the elasticity tended to decrease non-significantly to a value that was not significantly different from the value in controls. Figure 4A.

The wave dispersion coefficient was significantly different between the three groups (p = 0.02). Values were significantly lower in the obese group than in the control group (post-hoc P < 0.05)(1.21 [1.04–1.42] kPa; 1.53 [1.34–1.97] kPa, respectively). In the surgical group (1.55 [1.15–1.65] kPa), the wave dispersion coefficient had a value close to that in the control group, without significant differences relative to the control and high fat diet groups. Figure 4B.

When considering the histology results, the elasticity was positively correlated to the number of fibro-inflammatory complexes (r = 0.61, P = 0.0059) (Fig. 4C,D). The wave dispersion coefficient was significantly and negatively related to the number of fibro-inflammatory complexes (r = −0.52, P = 0.0216).

#### Fat fraction and R2*

Overall, the fat content measured at MR imaging from both groups was low. The obese group had a significantly higher pancreatic fat content than the control group (0.39 [0.00–1.59]% versus 0.00 [0.00–0.01]%, P < 0.05). The pancreatic fat content of the surgery group was intermediate (0.20 [0.00–0.49]%) and statistically indistinguishable from either the control or the obese groups. Pancreatic fat content determined with MR imaging was highly correlated to the number of adipocytes per unit area (r = 0.71, P = 0.0005, Fig. 4E).

The transverse relaxation rate R_2_* was significantly faster in the obese group than in the control group (104.39 [94.14–164.42] Hz vs. 83.92 [63.88–93.38] Hz, respectively), while the surgery group had R_2_* values of 83.80 [64.57–90.20], close to those observed in the control group. Values in the obese group were significantly higher than those obtained in the surgery (P < 0.001) and in the control (P < 0.001) groups, while values in the surgery group were not significantly different from those in the control group. A significant correlation was observed between R_2_* and the number of iron deposits (r = 0.77, P = 0.0001) (Fig. 4F).

#### Diffusion-weighted MR Imaging

The ADC was not significantly different between any group with values of 0.73 [0.57–0.79], 0.74 [0.63–0.95] and 0.73 [0.71–0.77], for the control, obese and surgery groups, respectively. No differences were found between animals displaying or not displaying intralobular adipocyte infiltration on pathological slides. No significant correlation was found between the ADC and the number of HHF.

**Figure 5:**
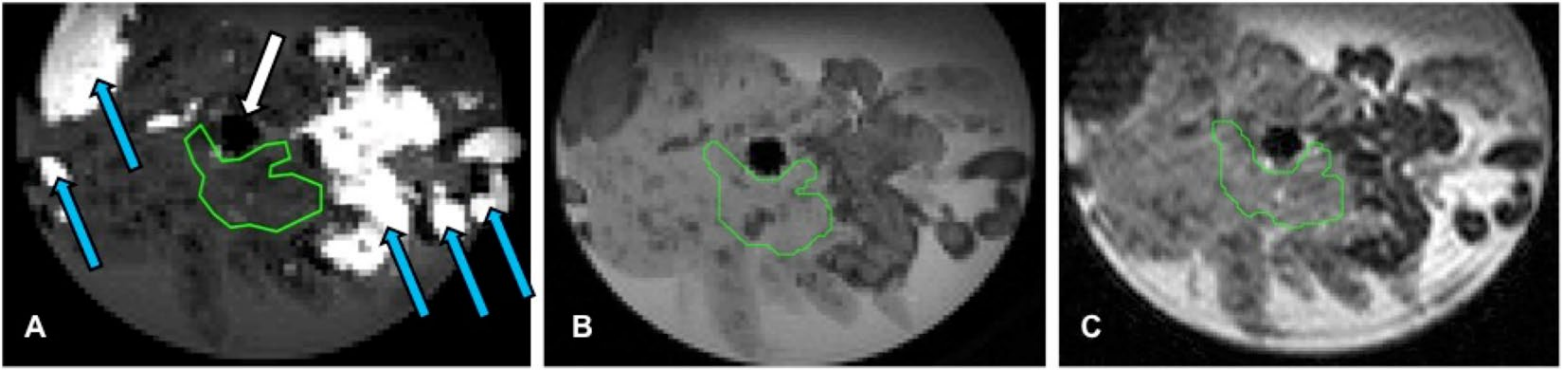
Representative images of a pancreas explant at MRI. These images are acquired in a horizontal plane across the circular sample holder, and form the basis for the computation of the quantitative MRI parameters described in the methods section. (**A**) Magnitude image obtained with the MR elastography sequence (spatial resolution: 300 µm, no filtering applied, repetition time: 1000 ms, echo time: 18 ms, flip angles: 90°−180°). The mechanical transducer is visible as a dark region at the center of the sample (white plain arrow). Pancreatic parenchyma appears as dark grey, whereas fatty tissue is visible as bright areas (blue arrows). The region of interest used for reporting quantitative results (yellow outline) is positioned close to the mechanical transducer and inside the pancreatic parenchyma. (**B**) Magnitude image obtained with the chemical shift-encoded gradient-echo MR imaging sequence (spatial resolution: 250 µm, no filtering applied repetition time: 950 ms, only a single echo time of 1.65 ms is displayed, flip angle: 15°). The region of interest (yellow outline) is co-registered to the region of interest selected for MR elastography. On this contrast, the pancreatic parenchyma appears light grey, whereas the fatty tissue appears as dark grey. (**C**) Magnitude image obtained with the diffusion weighted imaging sequence (spatial resolution: 195 µm, no filtering applied, repetition time: 2000ms, echo time: 20 ms, flip angles: 90°−180°, fat suppression applied). Only the first b-value (b = 0 s/mm²) is displayed. The region of interest (yellow outline) is co-registered to the region of interest selected for MR elastography. On this contrast, the pancreatic parenchyma appears as light grey, whereas the fatty tissue appears as very dark gray due to the fat suppression.

Representative images of a pancreas explant at MRI are showed in Fig. 5.

## Discussion

In this study, we assessed pancreatic lesions associated with obesity and high fat diet. Obese rats developed adipocyte infiltration and exocrine lesions of pancreatopathy characterized by acinar-ductal metaplasia, fibrosis, and iron deposits mainly located at the endocrine-exocrine interface. These lesions increased with the animal weight and duration of the diet, and could be assessed by MR imaging and elastography and partly reversed after bariatric surgery.

Obesity is the most common cause of insulin resistance and increases susceptibility for pancreatic disorders, such as pancreatitis and pancreatic adenocarcinoma, by involving low- grade chronic inflammation^9,22^. Even if the pathways between obesity and inflammation are not fully understood, several mechanisms have been proposed. The expansion of fat mass, due to adipocyte enlargement, increases the fat infiltration by macrophages, and the inflammatory M1-type, especially^8,23^. Obesity promotes macrophage recruitment and the secretion of numerous inflammation cytokines as TNFβ, IL-1, IL-6, and IL-18. These inflammatory mediators promote a chronic systemic inflammation in a paracrine manner. This study confirmed a direct link between pancreatic pathological changes and obesity. However the respective roles of the fat tissue, the high fat diet and the pancreatic fat infiltration in the pancreatic toxicity can be addressed to explain these changes on endocrine and exocrine tissue.

Animal models have shown that chronic high fat diets in rats induce fibrogenesis^24^. *In vitro* studies and mice models using high fat diets showed that lipid peroxidation products play a key role in stellate cell activation and fibrogenesis. Moreover, Philip *et al*. showed that HFD can activate oncogenic Kras via COX2, leading to pancreatic inflammation and fibrosis, and development of PanINs and pancreatic cancer in mice. Consumption of a HFD can be an inflammatory stimulus to trigger Kras signalling, initiating the development of cancer^25^.

The implications of pancreatic fat with reference to its proximity to pancreatic acinar cells and its toxic effects on acinar cells are still unknown. Fat adjacent to acinar tissue has been shown to be associated with the worst parenchymal damages immediately around the fat in acute pancreatitis in case of adipocyte necrosis^26^. However, the crosstalk between adipocytes, pancreatic stellate cells, endocrine, and exocrine tissue is still unknown.

In this study, fibrosis and acinar-ductal metaplasia were more prevalent in obese rats. ADM is first a part of regenerative process in the pancreas but in long term ADM is also considered as the first step of pancreatic oncogenesis. It is possible that these long-term changes modify the pancreatic environment with potential cancer risk. The main interest of this work is to show that these lesions could regress after surgery. Regarding the over risk of pancreatic cancer in obese patients, this point has to be specifically highlighted. As already published, this phenotype change is promoted by inflammation and pancreatic injuries. Our results are in keeping with the finding that obesity and pancreatic fatty infiltration are risk factors of PanIN lesions in humans. We previously reported that fatty infiltration, especially in intra lobular location, could promote precancerous lesions (OR = 17.86 [4.935–88.12]), independently of the age of the patients and the presence of diabetes^27^.

The histopathological studies of the pancreas of obese rats showed numerous and severe lesions involving islets and their surrounding exocrine parenchyma, that we called HHF, combining hypertrophy, hyper-vascularization and islets fibrosis. These features were correlated with glycoregulation abnormalities and increases in the rat weight. So both islet morphology and function are altered in obese rats. These islets characteristics have been partly observed in other models of obesity such as Zucker fatty rats or WNIN mutant rats^28,29^. Hence, it is assumed that the high fat-induced peripheral insulin resistance could promote a compensatory islet cell proliferation, followed by islet inflammation-mediated degeneration and fibrosis^30^. In a previous study, Schneider *et al*. showed that HFD induced an altered local insulin release and was associated with a significant increase in islet cell turnover and pancreatic cancer induction in rodents^31^. In our model, we did not find differences in insulin staining in b-cells in HFD rats and could not confirm the hypothesis discussed in recent mice models of diet-induced obesity^32^. However, as in the literature, we found an increase of the oral glucose tolerance test and insulin secretion after bariatric surgery^33,34^. Interestingly, we demonstrated that in our model, abnormal pancreatic features were detected not only in endocrine, but also in the exocrine pancreas, mainly observed beside HHF lesions, consisting in acinar-ductal metaplasia with inflammation, fibrosis, activation of stellate cells and iron deposits. Chronic inflammation and early precancerous lesions, locally favoured by both HHF and fatty infiltration, could account for higher risk of cancer in obese patients. Fibrosis and hypervascularization may induce local micro haemorrhage and iron overload that may amplify pancreatic damages.

The originality of our model is the evaluation of bariatric surgery as a potential treatment and prevention of fibrosis and precancerous lesions (acinar-ductal metaplasia). Lesions were indeed reversible. While bariatric surgery is one of the more efficient treatments of morbid obesity, few data are available in the literature to assess its consequences on systemic inflammatory profiles. It is also difficult to determine the precise effect of different bariatric surgical procedures on the beta cell function, particularly the early insulin secretion after stimulation by glucose, because of the considerable heterogeneity among studies^32^. No experimental data reported morphological changes of pancreatic lesions after surgery. Our results showed for the first time the impact of bariatric surgery on obesity-induced pancreatopathy, which is relevant for humans. In our series, no difference was found regarding morphological lesions according to the type of procedures (by pass or sleeve gastrectomy).

In this study, the pathological changes could be assessed with quantitative MR imaging. We observed significant changes of pancreatic mechanical properties, proton density fat fraction and R2* in obese rats. These changes paralleled the increases in HHF, adipocytes and iron deposits observed with obesity. Moreover, the changes in the MR and pathological features reversed significantly in parallel after surgery. Particularly, significant correlations were observed between pancreatic elasticity and wave scattering coefficient at MR elastography on one hand and the number of fibrotic lesions at pathology on the other. These results are in agreement with reported findings. Liver elasticity measurements at MR elastography are accurate markers of liver fibrosis stage in patients and animal models^16,35^. Recently, it has been reported that the wave scattering coefficient and the related “damping ratio” parameter at MR elastography are complementary markers of liver inflammation^36,37^.

Few elastography studies have been performed in the pancreas, mostly with ultrasound elastography. Increased stiffness values related to fibrosis and inflammation have been reported with ultrasound elastography in chronic and acute pancreatitis in clinical studies and in one mice study^11,38^. Such findings have not yet been reported with MR elastography but the feasibility and reproducibility for pancreatic MR elastography were reported in patients^10,39,40^.

We observed significant changes of proton density fat fraction and R2* at MR elastography in obese rats; these MR parameters were correlated to pancreatic fat and iron spots at histopathology. Obesity is associated with ectopic fat deposition in parenchyma organs such as pancreas^41^. Proton density fat fraction has been used to assess pancreatic fat in clinical studies^13,14^. As shown in our study, it is important to use T2* corrected MR sequences to assess PDFF in the pancreas because iron deposits (and the related R2* measurements) increase in obese rats^13,42^.

We did not observe significant changes in ADC in obese rats versus controls. These results are in agreement with the findings of Yoon JH *et al*. who observed a lack of ADC change related to pancreatic fibrosis in patients who had MR Imaging before surgical pancreatectomy^14^.

Our MR imaging study was performed *ex vivo*. The absence of perfusion in our model may have influenced the results of MR elastography and diffusion-weighted MR imaging^43^. The results have thus to be confirmed *in vivo* in future studies. Other MR sequences have also been proposed to stage pancreatic fibrosis, including intravoxel incoherent motion diffusion-weighted MR imaging^14^, kurtosis diffusion-weighted MR imaging^44^ and T1 relaxometry^45^. However, in the liver, these MR methods have not yet been shown to be more accurate than MR elastography to stage fibrosis^46^.

Several limitations could be addressed and the descriptive characteristics of the results especially. No mechanistic insight is proposed to understand the physiopathological processes. However, we confirmed that high fat diet induced obesity is a risk factor for pancreatopathy and inflammatory pancreatic lesions especially, both in acinar location and at the endocrine-exocrine interface and for early precancerous lesions. Bariatric surgery and obesity management can reverse these lesions. The results of our *ex vivo* MR imaging study suggest that the reversible changes in pancreatic fat, iron, and fibrosis in obese rats can be quantified with quantitative MR imaging using multi-echo gradient echo MR imaging and three-dimensional MR elastography. Applications in humans have to be developed in order to propose a new screening tool, especially to detect obese patients at high risk of pancreatic cancer.
